# Digital breast tomosynthesis image reconstruction using 2D and 3D total variation minimization

**DOI:** 10.1186/1475-925X-12-112

**Published:** 2013-10-31

**Authors:** Metin Ertas, Isa Yildirim, Mustafa Kamasak, Aydin Akan

**Affiliations:** 1 Electrical and Electronics Engineering Department, Istanbul University, Avcilar, 34320 Istanbul, Turkey; 2 Electrical and Electronics Engineering Department, Istanbul Technical University, Maslak, 34469 Istanbul, Turkey; 3 College of Engineering Department, University of Illinois at Chicago, Chicago, IL 60607, USA; 4 Computer Engineering Department, Istanbul Technical University, Maslak, 34469 Istanbul, Turkey

**Keywords:** Breast tomosynthesis, Compressed sensing, Total variation

## Abstract

**Background:**

Digital breast tomosynthesis (DBT) is an emerging imaging modality which produces three-dimensional radiographic images of breast. DBT reconstructs tomographic images from a limited view angle, thus data acquired from DBT is not sufficient enough to reconstruct an exact image. It was proven that a sparse image from a highly undersampled data can be reconstructed via compressed sensing (CS) techniques. This can be done by minimizing the l_1_ norm of the gradient of the image which can also be defined as total variation (TV) minimization. In tomosynthesis imaging problem, this idea was utilized by minimizing total variation of image reconstructed by algebraic reconstruction technique (ART). Previous studies have largely addressed 2-dimensional (2D) TV minimization and only few of them have mentioned 3-dimensional (3D) TV minimization. However, quantitative analysis of 2D and 3D TV minimization with ART in DBT imaging has not been studied.

**Methods:**

In this paper two different DBT image reconstruction algorithms with total variation minimization have been developed and a comprehensive quantitative analysis of these two methods and ART has been carried out: The first method is ART + TV_2D_ where TV is applied to each slice independently. The other method is ART + TV_3D_ in which TV is applied by formulating the minimization problem 3D considering all slices.

**Results:**

A 3D phantom which roughly simulates a breast tomosynthesis image was designed to evaluate the performance of the methods both quantitatively and qualitatively in the sense of visual assessment, structural similarity (SSIM), root means square error (RMSE) of a specific layer of interest (LOI) and total error values. Both methods show superior results in reducing out-of-focus slice blur compared to ART.

**Conclusions:**

Computer simulations show that ART + TV_3D_ method substantially enhances the reconstructed image with fewer artifacts and smaller error rates than the other two algorithms under the same configuration and parameters and it provides faster convergence rate.

## Background

Several imaging modalities such as mammography, ultrasound and magnetic resonance have been extensively used in breast imaging for decades. Digital breast tomosynthesis (DBT) is an imaging technique using limited range of view angles, hence it has limitations in implementation and challenges in image reconstruction [1]. Recently, digital tomosynthesis has become an emerging imaging modality for breast imaging where the breast is projected onto a flat panel detector from a limited view angle and a few number of projections is acquired to produce a three dimensional image of the breast. It is now currently being used for both diagnosis and screening purposes [1]. This technique overcomes the overlapping problem which is the most present artifact in 2D mammographic imaging. However, due to the limited range of view angles, the number of projections may not be sufficient to fully reconstruct the image in the Fourier space [2]. More specifically, the Fourier space is not fully sampled which causes streaking artifacts. In order to acquire tomosynthesis images, algebraic reconstruction technique (ART) is adapted to acquire 3D objects from two dimensional projection images [3]. Although several studies has shown that iterative algorithms can show satisfactory results over filtered back projection (FBP), modified FBP and matrix-inversion algorithms in missing data image reconstruction, they still do not give acceptable results in tomosynthesis imaging [2,4,5].

DBT image reconstruction algorithms have shown significant improvements over the years. Shift and add (SAA) which is based on shifting and adding projections to sharpen the plane focus was the first idea of DBT image reconstructions [6]. However, significant amount of out of focus slice blur occurs in SAA. FBP, modified FBP and iterative methods have also been widely used in DBT image reconstruction perspective [7-9] to overcome the blurring problem. Among these techniques, iterative methods are generally suitable for complete data sets or nearly complete data sets. However, in tomosynthesis, acquired data is highly incomplete thus acquiring the exact image can be severely affected by this incompleteness. This problem can be addressed in a compressed sensing (CS) framework which allows the reconstruction of a signal or image from a highly undersampled observation [10]. Hence after introduction of the CS approach, the number of studies addressing sparse image reconstruction has drastically increased.

Iterative methods and FBP methods are not sufficient enough to preserve the edges which play an important role in identifying objects and fundamental features in the image. However, total variation (TV) minimization was proven to be applicable in edge preserving image denoising processes [11,12]. This algorithm is based on minimizing the l_1_ norm of the sparsified image. TV minimization has recently been adapted to DBT imaging reconstruction problems and better results were obtained than FBP and iterative methods [13,14]. By adding different constraints, such as prior image constrained CS, improved results were obtained over CS and FBP algorithms [13]. Moreover, compressed imaging based on wavelet sparsity has been investigated in limited tomographic x-ray imaging [15] and MRI [16] to reconstruct the image from fewer projections and promising results have been obtained. Another alternative approach was introduced in [17] where, curvelets were used for reconstruction at a limited angular range and the results showed that the method was stable and edge-preserving.

Previous studies of TV minimization have shown significant improvement in various imaging problems [11-14,18-20]. Most of them have addressed 2D image reconstruction [11-13,18] and a few of them have shown results on TV_3D_ regularization [14,19,20]. Though both TV_2D_ and TV_3D_ minimizations have been studied as separate works, neither of those studies has addressed if TV_3D_ minimization has a superiority over TV_2D_ minimization on limited view angle 3D image reconstruction. In the present work, two different DBT image reconstruction algorithms with total variation minimization have been developed and a comprehensive quantitative analysis of these two methods and ART has been carried out:

i) ART + TV_2D_ method: TV_2D_ was implemented layer by layer along the axial dimension to fully cover the entire image.

ii) ART + TV_3D_ method: TV_3D_ was implemented to the entire space to get a reconstructed image in a single step.

A specific 3D phantom which roughly simulates a breast tomosynthesis image was designed to compare the reconstruction performances of ART, ART + TV_2D_ and ART + TV_3D_ methods in the sense of root mean square errors (RMSE) of reconstructed 3D image and a specific layer of interest (LOI). The visual perception assessment was done by means of structural similarity (SSIM) curves.

### Reconstruction methods

In linear imaging problems, the following model is used:

(1) Y=AX+n,

where Y shows the measured or observed data, X is the original image, A shows the system matrix which gives the data measurement process in the image and n is the additive noise. Consistency condition in (1) should be fulfilled in image reconstruction algorithms. Image reconstruction is an inverse problem which is based on estimation of X from the given Y and A. In (1), the system matrix may vary according to the imaging problem such as Fourier transform in MRI or Radon transform in tomographic reconstruction.

### Algebraic reconstruction technique (ART)

In tomosynthesis imaging, limited view angle creates a large portion of missing data which makes the exact image reconstruction more difficult. Thus, iterative image reconstruction techniques are introduced to estimate the exact object. ART is one of the most commonly used iterative reconstruction technique in image processing [3,21,22]. In ART, an image is estimated in an iterative manner while satisfying the consistency condition in (1). The idea that lays behind the reconstruction is that the voxel intensities are updated ray by ray for each projection. A 3D image is updated with ART reconstruction by using the following formulation:

(2) $$X_{j}^{\left(k+1\right)}=X_{j}^{\left(k\right)}+\frac{\left(Y_{i}-\sum_{k=1}^{N}A_{\mathrm{ik}}.X_{j}^{\left(k\right)}\right)}{\sum_{k=1}^{N}A_{\mathrm{ik}}^{2}}A_{\mathrm{ij}},\begin{array}{c} j=1,2,\ldots ,N\\ i=1,2,\ldots ,M \end{array},$$

where Y shows the measured detector data, X is the image to be reconstructed, i and j are ray and voxel indexes respectively. M is the total number of rays and N shows the number of voxels. A, the system matrix, is formed by computing the length of the intersection segment of each voxel on each ray for all projections. Calculating the ray path for the 3D reconstruction is done by using Siddon’s algorithm [23]. The difference between the measured and calculated projection data is backprojected along each ray of the related projection based on the Siddon’s coefficients. Error backprojection procedure is continued for all projections to finish one iteration. This iteration process is repeated until a convergence criterion is satisfied. However, the measured projection data in DBT is not sufficient enough to reconstruct an exact image due to the limited view angle.

### Total variation (TV) minimization

Out-of-focus slice blur and streaking artifacts arise as a result of missing data in DBT. Therefore, in order to improve the quality of image acquired from highly undersampled data, improved methods are needed. It was proven that a sparse image can be recovered from a highly undersampled data set which is called compressed sensing (CS) [10]. The compressed sensing can be represented as minimization of l_1_ norm of the sparsified image X:

(3) $$\min\;|\;\mathrm{\Psi X}|{}_{1}\;\mathrm{such}\;\mathrm{that}\;Y=\mathrm{AX},$$

where Ψ represents a linear operator called the sparsifying transform. Discrete gradient and wavelet transforms are the most commonly used sparsifying transforms in CS theory. In this study, discrete gradient transform is used and applied in two different forms as 2D and 3D. Considering the discrete gradients for each pixel in the image, the problem turns into minimizing the TV(X). Thus, TV of a 2D image can be shown as:

(4) $$TV_{2D}\left(\;X_{\left(i,j\right)}\right)=\sum_{i=1}^{K}\sum_{j=1}^{L}\sqrt{|X_{\left(i,j\right)}-X_{\left(i+1,j\right)}{|}^{2}+|X_{\left(i,j\right)}-X_{\left(i,j+1\right)}{|}^{2\;}}$$

where, X_(i,j)_ is the intensity value at pixel (i,j), {i = 1,2,…..,K; j = 1,2,……,L}. Applying TV_2D_ layer by layer to a 3D image shows significant improvements in image quality [24]. But applying the TV term in such manner ignores the neighborhood of a 3D image along axial direction. Considering this neighborhood relation, the same discrete gradient transform may also be adapted in 3D form. TV of a 3D image can be shown as:

(5) $$TV_{3D}\left(X_{\left(i,j,k\right)}\right)=\sum_{i=1}^{K}\sum_{j=1}^{L}\sum_{k=1}^{M}\sqrt{{\left(D_{x}X\right)}^{2}+{\left(D_{y}X\right)}^{2}+{\left(D_{z}X\right)}^{2}}.$$

where X_(i,j,k)_ is the intensity value at voxel (i,j,k), {i = 1,2,…,K, j = 1,2,…..,L and k = 1,2,…..,M}. (*D*_*x*_*X*) = *X*_(*i*,*j*,*k*)_  − *X*_(*i* + 1,*j*,*k*)_, (*D*_*y*_*X*) = *X*_(*i*,*j*,*k*)_  − *X*_(*i*,*j* + 1,*k*)_, (*D*_*z*_*X*)*X*_(*i*,*j*,*k*)_  = *X*_(*i*,*j*,*k* + 1)_. Implementing TV minimization as a penalty term has been shown to give improved results [2,6,12-14]. Thus, a widely used CS-based constrained minimization problem has been formulated as:

(6) $$\mathrm{mi}n_{X}\left[\;∥\nabla )X∥{}_{1})\right],\mathrm{such}\;\mathrm{that}\;\mathrm{AX}=Y.$$

This constrained minimization problem can be reformulated as an unconstrained minimization problem as:

(7) $$\mathrm{mi}n_{X}\left[∥Y)-\mathrm{AX}∥{}_{2})+\alpha ∥\nabla )X∥{}_{1})\right],$$

where, α is the regularization parameter which regulates the effect of TV inclusion to the objective function. In this work, the reconstruction is performed as two different parts:where $\hat{X}$ is reconstructed image by ART.

1. Applying ART to create the 3D image: Reconstructed image is acquired applying the following steps:

a. An initial image is selected or assigned.

a. System matrix in (1) is calculated using the Siddon’s ray-driven algorithm.

a. The measured detector data and the forward projection of the image are compared.

a. New image is obtained using ART (2).

2. Total variation minimization: (8) is minimized in order to acquire total variation minimized of image reconstructed by ART:

(8) $$\mathrm{mi}n_{X}\left[∥X)-\hat{X}∥{}_{2})+\alpha ∥\nabla )X∥{}_{1})\right],$$

#### Initialization

Given *N*_*i*_*, N*_*r*_*, N*_*p*_*, α, Ԑ*_*o*_*, S*_*(x,y,z)*_*, D*_*(x,y,z)*_*,D*_*s*_*, Y*

$$X_{k}^{p}=\mathrm{Zeroes}\;\mathrm{initial}$$

#### System Matrix Calculation

#### Reconstruction Algorithm Step

*for ite = 1,2,3.....,N*_*i*_

#### Art using (2)

#### TV Minimization using (8) for TV _3D_

The pseudo code for the ART + TV_3D_ algorithm is shown in Algorithm of ART + TV_3D_ reconstruction where S_(x,y,z)_ and D_(x,y,z)_ represent the coordinates of X-ray source and detector, D_s_ is the size of the detector, N_i_, N_p_, N_r_, N_v_ show number of iterations, projections, rays and voxels respectively and k is the voxel coordinate. Measured data is shown as Y. *α* is the regularization parameter in TV minimization step and ϵ_o_ shows the value for stopping criterion of TV. By using this algorithm, in the present work, two different TV methods were investigated called as TV_2D_ and TV_3D_. In the first method, TV_2D_ inclusion is applied layer by layer to cover the entire image. In the second method, in order to apply TV_3D_ into ART, TV minimization step is applied to the entire phantom at once after the reconstructed image obtained at each iteration. There are several factors in the algorithm which may increase the speed of convergence and the efficiency of the optimization problem. The first one is the initial guess of the image. Prior image constrained algorithm was conducted better results than classical ART, CS and FBP [13]. However the image registration needs to be taken into account in order to achieve a faster convergence speed. In this study, the initial image is chosen to be zeros. The second point is the stopping criterion of the iterative steps. The iteration is continued until the difference between two consecutive updated images is smaller than a certain value. The last item is to choose the appropriate regularization parameter in the objective function (8) which plays a critical role in the performance of TV minimization [24].

## Results

TV_2D_ and TV_3D_ have been extensively used in various medical imaging problems [11-14,18-20]. Most of the studies have addressed TV_2D_ in minimizing the total variation and only few studies have addressed TV in 3D manner. However their performance comparison has not been fully investigated. In our study, in order to observe the performance of three reconstruction algorithms used in DBT, a 3D phantom data was created and a tomosynthesis system was designed.

In the commercial DBT modalities, characteristics of DBT systems show differences in terms of several parameters, such as detector motion, X-ray tube motion, angular range, number of projections, source-detector distance, detector to the center of rotation distance, reconstruction methods and so on [25]. Parameters of the designed system and phantom are shown in Table 1. One of the most important problems which directly affects the reconstruction quality in limited-view angle imaging is the angular range θ. In this study a range of 50^0^ is considered with a scanning θ from -25^0^ to +25^0^. The number of projection is limited to 11 as the increment of θ is 5^0^ for each projection. A single X-ray source and a virtual detector with a size of 81*81 pixels are used for the simulations. X-ray tube motion is step-and-shoot with a circular rotating detector.

**Table 1:** Simulation parameters

**Parameter**	**Value**
Detector motion	Rotating
X-ray tube motion	Step-and-shoot
Source to detector distance	300 pixels
Object to detector distance	50 pixels
Angular range (deg)	50° degrees (−25° to +25°)
Number of projections	11 projections
Reconstruction method	Iterative with TV regularization
TV regularization parameter *α*	0.8
Number of iteration	60
Phantom size	71*71*10
Detector size	81*81

Phantom with a size of 71*71*10 was specifically designed to demonstrate the most common overlapping tissue problem in tomosynthesis imaging. In this study, since our aim is to study if ART + TV_3D_ has a superiority over ART + TV_2D_ on limited view angle 3D image reconstruction in terms of reconstruction quality, the size of the phantom and the detector matrix we use are only chosen for the simplicity and to speed up the simulations.

**Figure 1:**
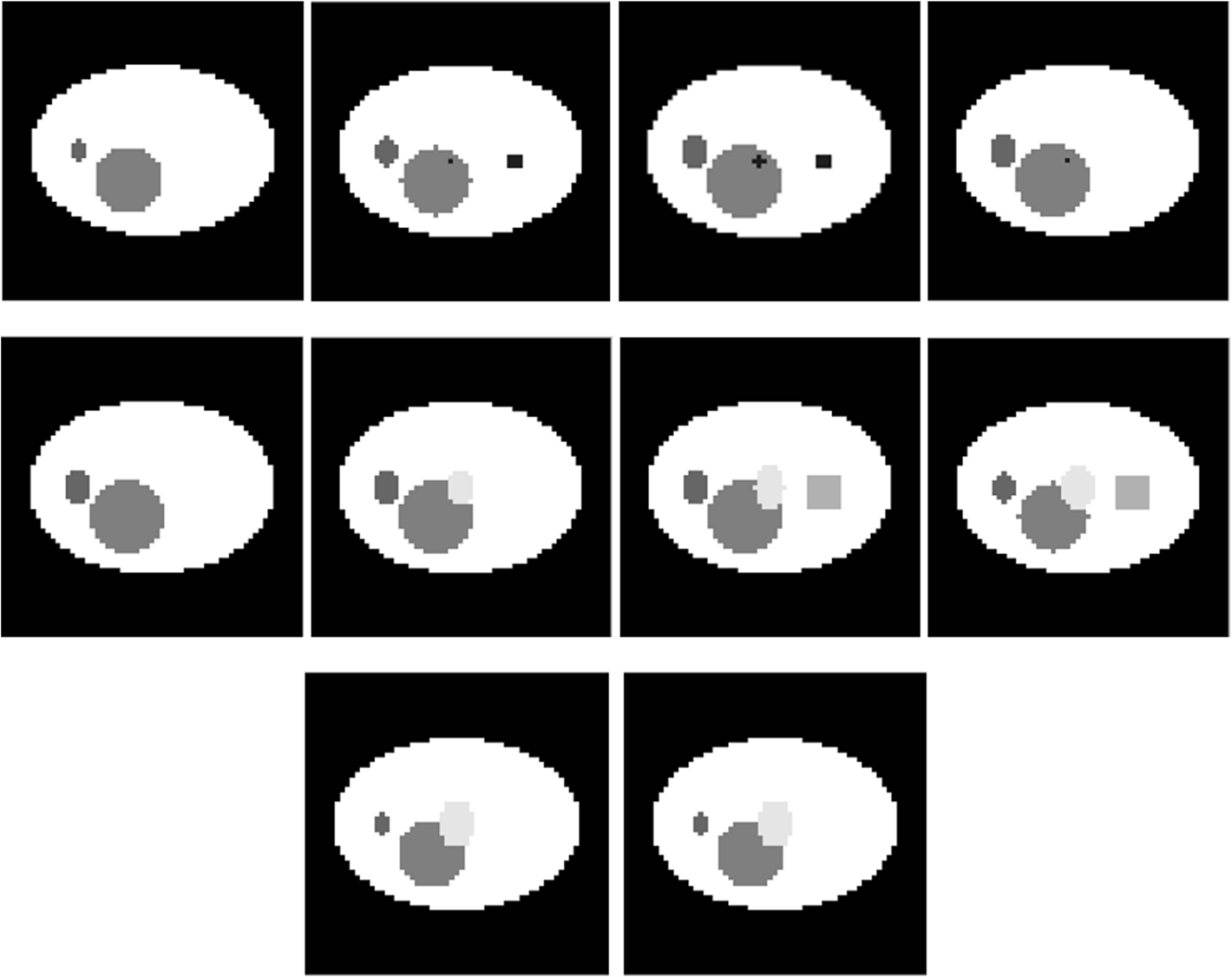
Phantom layers (top left to right: 1st layer, 2nd layer, 3rd layer (LOI), 4th layer, 2nd row from left to right: 5th layer, 6th layer, 7th layer, 8th layer; 3rd row from left to right: 9th layer 10th layer).

The phantom consists of 10 layers in axial dimension which gives a closer match with real tomosynthesis imaging. There are some smaller objects with lower x-ray absorption rates located in the lower slices of the phantom. These small objects are obscured by objects with high x-ray absorbance in the upper slices of the phantom. 3rd layer of the image was chosen to be the LOI of the image due to its structure for the possible screening problem in 2D mammography imaging. The small square in the right side of the LOI represents an object with a very low x-ray absorption rate. In order to imitate the screening effect in 2D imaging a larger object with a higher x-ray absorption rate was located in the layers 7 and 8. Objects in the phantom or the phantom itself can be modified and extended for different purposes. The layers of the phantom used in the study are demonstrated in Figure 1.

In DBT imaging the out-of-focus-slice blur in the layer of interest is the most dominant artifact, thus the simulations are performed with noiseless projection data. The same reconstruction parameters have been selected for ART, ART + TV_2D_ and ART + TV_3D_ methods. An experience-based fixed regularization parameter *α* is set to 0.8 for ART + TV_2D_ and ART + TV_3D_ methods in our experiments. All simulations were performed in MATLAB® software. The system configurations which are used for the simulations are Intel(RM) Core(TM) i7-2630 QM CPU @ 2.00 GHz CPU, 6 GB Memory, Windows 7 64 Bits operating system.

Performances of three reconstruction algorithms were compared both qualitatively and quantitatively. For qualitative assessment, the visual observation was conducted and the structure similarity value which shows the visual quality was used. For quantitative assessment, RMSE values of the LOI and 3D image were compared.

**Figure 2:**
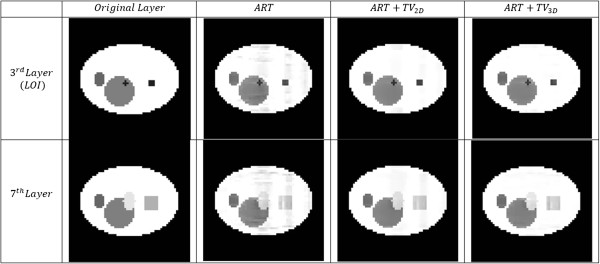
Results of the reconstruction algorithms for the LOI and 7th layer.

In this study the projections were acquired from 11 view angles and the reconstruction algorithms were performed for 60 iterations. Figure 2 shows the results of the reconstruction algorithms for the LOI and 7th layer. Second column shows images reconstructed by ART. Compared to the original image the blur in images reconstructed with ART is apparent and in the 7th layer visible distortion makes the reading difficult. Compared with the reconstructed image by ART, reconstructed images using ART + TV_2D_ and ART + TV_3D_ (the third and fourth columns respectively) are significantly improved. Both ART + TV_2D_ and ART + TV_3D_ methods preserve the edges, however it is clearly seen in images reconstructed with ART + TV_2D_ that the blur is still existent. In images reconstructed with ART + TV_3D_ the noise is lower than the other two methods. ART + TV_3D_ reconstructs images closer to the original images than ART and ART + TV_2D_.

**Figure 3:**
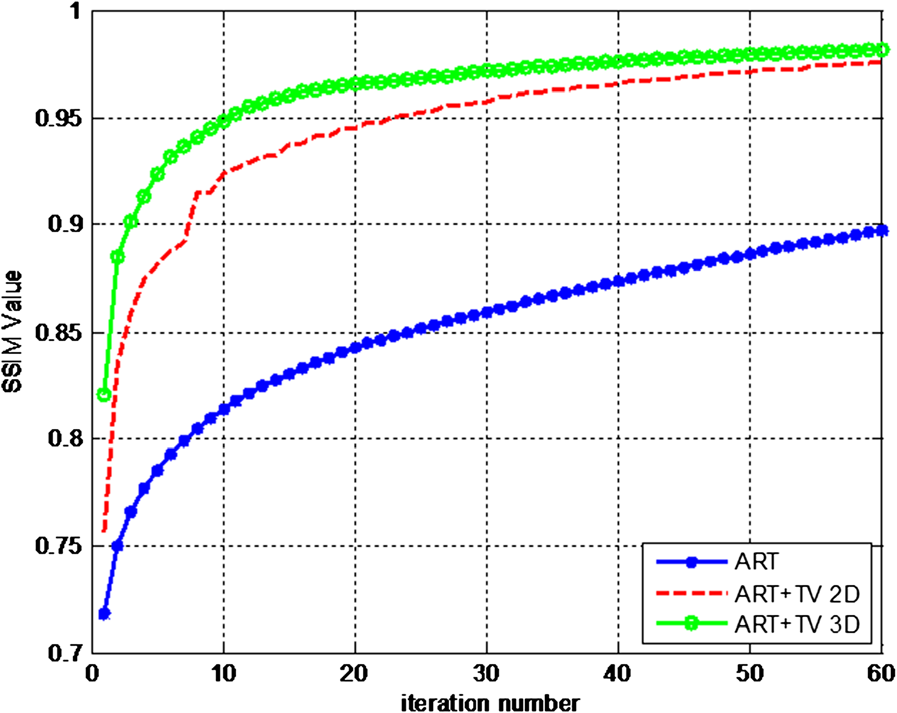
MSSIM graph of 3rd layer (LOI).

Since the human visual perception is highly adapted for extracting structural information from images it was introduced an alternative complementary framework for quality assessment based on the structure similarity (SSIM) between two images [26]. For image quality assessment, SSIM index is used locally rather than globally. Thus, parameters used to calculate the SSIM are computed within a local window which moves pixel by pixel through the image. For a single overall image quality index between two images the following equation is used:

(10) $$\mathrm{MSSIM}\left(X,Y\right)=\frac{1}{M}\sum_{i=1}^{M}\mathrm{SSIM}\left(x_{i},y_{i}\right),$$

where, X and Y are the reference image and reconstructed image respectively; x_i_ and y_i_ are the image index at the i-th local window in X and Y respectively. M shows the number of local windows. This equation calculates mean of SSIM values in all local windows. MSSIM values show much better consistency with the qualitative results. MSSIM values of the three reconstruction methods for the LOI are shown in Figure 3.

**Figure 4:**
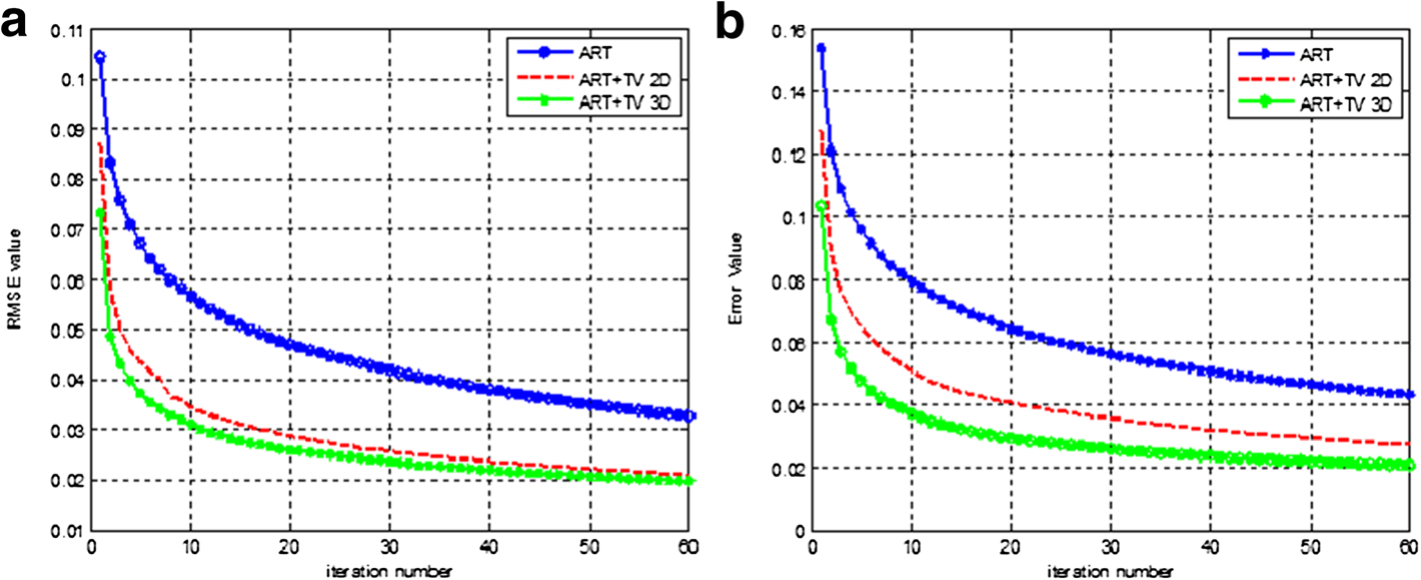
**RMSE performance comparison of the reconstruction algorithms a) RMSE graph of 3rd layer (LOI). b)** Total RMSE graph.

The best similarity is provided between reconstructed image and reference image at the value of 1. The overall SSIM values at 60th iteration are 0.8973, 0.9765, 0.9816 for ART, ART + TV_2D_ and ART + TV_3D_ respectively. In this sense, SSIM values for ART + TV_2D_ and ART + TV_3D_ show superior results compared to the ART reconstruction. Although the values for ART + TV_2D_ and ART + TV_3D_ are very similar to each other, the convergence speed of ART + TV_3D_ is faster than ART + TV_2D_. ART + TV_3D_ reaches to the value of 0.955 in 12th iteration while ART + TV_2D_ can reach to that value in the 27th iteration. Thus, ART + TV_3D_ shows better results than the other two reconstruction methods. Also notice that the SSIM curve for ART + TV_2D_ shows fluctuations unlike the other two reconstruction methods because of the absence of neighborhood relationship in axial dimension. These fluctuations can also be seen for ART + TV_2D_ in Figure 4a. Comparison of RMSE values of the methods for the LOI is shown in Figure 4a. The RMSE values at 60th iteration are 0.0327, 0.0212, 0.0198 for ART, ART + TV_2D_ and ART + TV_3D_ respectively. ART + TV_3D_ gives better RMSE performance than ART + TV_2D_ and much better RMSE performance than ART.

For further performance analysis of the methods, the total RMSE values of ART, ART + TV_2D_ and ART + TV_3D_ are compared in Figure 4b. ART shows the highest error value when it is compared to the other two methods. After 60th iteration, the error value for ART is 0.0433. ART + TV_2D_ gives lower error value than ART with the error value at 60th iteration 0.0273. ART + TV_3D_ shows the best performance as giving 0.0206 total RMSE value at 60th iteration. The error difference between ART + TV_2D_ and ART + TV_3D_ is slightly closer in Figure 4a than in Figure 4b. Increasing the number of layers in axial direction may also increase the gap in performances of these two reconstruction methods.

Though both ART + TV_2D_ and ART + TV_3D_ methods improved the results of ART considerably, ART + TV_3D_ is preferred over ART + TV_2D_ due to its faster convergence rate. Reaching an error value of 0.043 for ART can only be achieved at 60th iteration. The same value is achieved for ART + TV_2D_ at 16th iteration and for ART + TV_3D_ at 6th iteration.

The computational cost of ART is considerably much more than analytic image reconstruction methods. Thus, improving the speed and effectiveness of ART is crucial while keeping the computational time in reasonable levels. In this study, the reconstruction time spent for a single iteration of ART + TV_3D_ is %1.8 more than the time spent for a single iteration of ART. Thus, implementing TV_2D_ or TV_3D_ with ART does not dramatically increase the time spent for the reconstructions while reducing the out of focus slice blur substantially.

## Conclusion

Breast tomosynthesis imaging problem has been studied by implementing CS into ART in two different manners: 1) ART + TV_2D_: TV minimization step was done by applying TV layer by layer in 2D form along the axial dimension, 2) ART + TV_3D_: implementation of TV in 3D form to fully cover the phantom in a single step. The numerical results were conducted to compare the performances of ART, ART + TV_2D_, ART + TV_3D_ by designing a breast phantom to simulate the overlapping tissue problem in breast tomosynthesis imaging. Results of this study demonstrated that including TV after ART in the reconstruction algorithm significantly reduced out-of-focus slice blur in the reconstructed images compared to ART. ART + TV_3D_ provided better results than two other reconstruction methods both quantitatively by giving smaller RMSE values of the LOI and 3D images and qualitatively by generating higher MSSIM values. The computational costs per iteration for the tested methods were almost the same due to the simplicity of total variation minimization step. However, ART + TV_3D_ provided the fastest convergence rate among all three methods. In conclusion, in tomosynthesis imaging due to high amount of missing data, improved reconstruction techniques need to be developed to have better reconstructed images. In this paper, it was shown that a 3D breast tomosynthesis image can be reconstructed much faster and less artifact-free with ART + TV_3D_ method than ART and ART + TV_2D_ methods.

## Abbreviations

DBT: Digital breast tomosynthesis; CS: Compressed sensing; TV: Total variation; ART: Algebraic reconstruction technique; 2D: 2 Dimensional; 3D: 3 Dimensional; ART + TV2D: Algebraic reconstruction technique with 2 dimensional total variation; ART + TV3D: Algebraic reconstruction technique with 3 dimensional total variation; SSIM: Structure similarity; RMSE: Root mean squared error; LOI: Layer of interest; FBP: Filtered back projection; SAA: Shift and add; MRI: Magnetic resonance imaging; MSSIM: Modified structure similarity; TV2D: 2 Dimensional total variation; TV3D: 3 Dimens0069onal total variation.

## Competing interest

The authors declare that they have no competing interests.

## Authors’ contribution

ME carried out the reconstruction simulations, performed analysis of the simulation results and drafted the manuscript. IY conceived of the study, participated in the design of the study, and helped in drafting the manuscript. MK participated in the design of phantom and system, and helped in drafting the manuscript. AA participated in the coordination and helped in drafting the manuscript. All authors read and approved the final manuscript.
